# Internet-Related Disorders and Their Effects on Personality Development in Adolescents from Germany—Results from a Prospective Study

**DOI:** 10.3390/ijerph19010529

**Published:** 2022-01-04

**Authors:** Kai W. Müller, Manfred E. Beutel, Leonard Reinecke, Michael Dreier, Christian Schemer, Mathias Weber, Anna Schnauber-Stockmann, Birgit Stark, Oliver Quiring, Klaus Wölfling

**Affiliations:** 1 Outpatient Clinic for Behavioral Addiction, Department of Psychosomatic Medicine and Psychotherapy, University Medical Center, Johannes Gutenberg University Mainz, 55131 Mainz, Germany; Manfred.beutel@unimedizin-mainz.de (M.E.B.); michael.dreier@uni-mainz.de (M.D.); woelfling@uni-mainz.de (K.W.); 2 Department of Communication, Johannes Gutenberg University Mainz, 55128 Mainz, Germany; reineckl@uni-mainz.de (L.R.); schemer@uni-mainz.de (C.S.); mathias.weber@uni-mainz.de (M.W.); anna.schnauber-stockmann@uni-mainz.de (A.S.-S.); birgit.stark@uni-mainz.de (B.S.); quiring@uni-mainz.de (O.Q.)

**Keywords:** Internet-related disorder, longitudinal study, mean-level changes, personality, trait-pathology associations

## Abstract

Internet-related disorders (IRD) are increasingly becoming a major health issue. IRD are defined as the predominant use of online content, related to a loss of control and continued use despite negative consequences. Despite findings from cross-sectional studies, the causality of pathways accelerating the development of IRD are unclear. While etiological models emphasize the role of personality as risk factor, mutual influences between IRD and personality have not been examined. A prospective study with two assessments was conducted with *n* = 941 adolescents (mean age of 13.1 years; 10–17 years). Our aim was to validate etiological assumptions and to examine the effects of IRD-symptoms on the maturation of personality. IRD were measured with the Scale of the Assessment of Internet and Computer game Addiction (AICA-S). Personality traits were assessed using the Brief Five Factor Inventory (BFI). Conscientiousness and neuroticism were predictive for IRD symptoms one year later, and were likewise prone to changes depending on incidence or remission of IRD. Conscientiousness and openness moderated the course of IRD symptoms. Our findings point to complex trait–pathology associations. Personality influences the risk of development and maintenance of IRD symptoms and pre-existing IRD-symptoms affect the development of personality. Adaptations to etiological models are discussed and perspectives for novel intervention strategies are suggested.

## 1. Introduction

Adolescence is a period of transition and crucial changes in many respects. The adolescent faces, not only biological, but also psychological and social adaptation. Not surprisingly, research has demonstrated that this period can be associated with elevated levels of distress. Some adolescents are prone to develop conduct problems with girls being vulnerable for internalizing and boys for externalizing mental problems [1,2]. According to developmental psychopathology these problems can be limited to the transitional process in adolescence which means that recovery from mental problems is likely. However, there is also evidence that occurring mental problems might persist to subsequent developmental periods. In other words, these symptoms outlast the transition from adolescence to early adulthood causing persistent problems for the individual and his social environment [3,4].

The excessive and poorly controlled engagement in Internet activities such as online computer games or social media has increasingly become a matter of scientific and clinical interest during the past few decades [5,6]. There is ongoing debate [7,8] of which online activities can be defined among the umbrella term “Internet-related disorders” (IRD; also referred to as Internet addiction). In 2013, “Internet Gaming Disorder” a well-studied subtype of IRD was included as a preliminary diagnosis (section III) of the DSM-5 [9]. Six years later, the World Health Organization [10] decided to include “Gaming Disorder” (GD) as a full diagnosis into ICD-11 where it will be subsumed among the chapter of “substance use and addictive behaviors”. Yet, a growing body of evidence has emerged showing that also further subtyped of IRD (e.g., social media disorder; addictive use of online-pornography) can be related to addictive behavior and display similar nosological characteristics [11,12]. On the other hand, these potential further types will not explicitly recognized in the ICD-11 and indeed some researchers have argued that they might be better classified as an obsessive-compulsive disorder [13].

Empirical investigations on GD and other forms of IRD grew rapidly and yielded important insights into crucial aspects of this novel mental health issue. Epidemiological studies have reported substantial prevalence rates, particularly among minors. For example, Müller et al. [14] examined about 13,000 adolescents from seven European countries and showed that 1.6% met criteria for GD with another 5.1% being at risk for GD. In a meta-analysis on IRD, Cheng and Li [15] prevalence rates for adolescents worldwide ranged between 5.1–6.9%.

Unfortunately, there is a current lack on prospective studies. One of the few exemptions is a longitudinal study by Wartberg et al. [16] who addressed Internet Gaming Disorder in a 1-year follow-up study on a sample of 1095 adolescents. The authors found that low self-esteem and hyperactivity were causal predictors of Internet Gaming Disorder one year later.

While there is good evidence for the prevalence and correlates of GD and IRD from cross-sectional studies, less is known about the causality of these associations. Thus, our knowledge on etiological factors and their contribution to the manifestation of GD and IRD is limited. Etiological models like for example, the Interaction of Person–Affect–Cognition–Execution model [17] or the Integrative Process Model of Internet Addiction (InPrIA); [18] postulate that personality traits influence the effects of exposition to specific online contents and the subsequent transition to excessive use and manifestation of addictive symptoms. At present, there are numerous studies on the associations between personality and GD and IRD [19,20,21,22,23] including one meta-analysis [24]. The latter reported the strongest associations with conscientiousness, neuroticism, and agreeableness. However, these findings have only been based on cross-sectional studies, so we cannot be sure if the direction of the pathways postulated are valid.

Clinical research on the effects of specific personality traits on mental disorders is complex and generally has indicated bidirectional associations. Contemporary models of personality espouse that personality traits are disposed to changes throughout lifetime [25]. They further assume that mean-level-changes are not occurring randomly but rather follow general patterns in terms of a functional maturation of personality [25]. Furthermore, it has been demonstrated that these changes are induced by both, biological and learning processes. The latter can be promoted by certain life events [26]. Similarly, maturing of personality can be affected by mental disorders, such as substance-use disorders [27]. For example, Hicks et al. [28] showed in a longitudinal study on adolescents that there were significant differences in personality development related to different patterns of alcohol consumption. Participants who had started drinking in a problematic way at early stages and who were still misusing alcohol as young adults displayed a slower maturation of personality than participants without problematic drinking behavior.

Thus, for the study on GD and IRD, two things are important: First, as personality is age-related, comparisons of individuals with vs. without IRD should control for age. Generally, subjects with GD/IRD are of younger age which leads to confounding results when comparing personality traits in cross-sectional studies. Secondly, there is reason to assume that traits are directly influenced by persisting symptoms of GD/IRD as it is the case in other mental disorders [28]. While findings from cross-sectional studies have repeatedly shown that especially high neuroticism is related to IRD, the question remains if high neuroticism can be regarded as a causal factor for developing IRD or if the natural maturing of this factor is impaired by IRD.

In order to provide first evidence on the relatedness between personality and IRD, we conducted a prospective study in German adolescents. Our main interest was to identify pathways between specific personality traits from the five-factor model of personality and symptoms of IRD. To that purpose, we tested different assumptions: (1) personality traits as risk factors for IRD; (2) personality traits as moderating factors; and (3) effects of IRD symptoms on the maturing of personality traits over the course of time (mean-level-changes). This led to the following hypotheses:

(1) According to previous findings, we expect that personality will generally be prone to changes in our sample of adolescents. However, since we are looking at a short time period, we expect changes to be of small effects sizes.
(2) Derived from previous findings on trait-pathology-associations, we expect that the development of personality traits will be influenced by IRD. Thus, following recommendations of Durbin and Hicks [27], personality traits are addressed as dependent variables. As this has not been investigated before for IRD, it is difficult to provide specific hypotheses. However, taking the findings of Hicks et al. [28] into account, we expect increases in neuroticism and decreases in extraversion and conscientiousness among adolescents with IRD.
(3) As an additional and exploratory approach, we also investigated personality as moderating factors. Derived from prior results of cross-sectional studies [24], we suppose that changes in neuroticism and conscientiousness will affect the stability of IRD- symptoms over one year.
(4) Based on findings from cross-sectional studies [24], we expect that personality traits, particularly high neuroticism and low conscientiousness at baseline will be predictive of IRD one year later.

Since there is evidence [12] pointing to gender-specific aspects regarding the development, manifestation, and expression of IRD, we controlled for this variable in each analyses.

## 2. Materials and Methods

### 2.1. Study Design and Participants

Starting in 2016, a representative sample of German adolescents was drawn from the state Rhineland-Palatinate. The questionnaire-based study “always on”, funded by the Research Center for Media Convergence of the local university, aimed to identify links between different variables of relevance for the development of use habits of new media. To that purpose, a probability sample of *n* = 1384 adolescents (aged 10–17 years; M = 13.1, SD = 1.16) was recruited and prospectively re-assessed in an interval of 1.3 years. To that purpose, each participant created an individual code when filling in the baseline questionnaire. This code followed standardized rules enabling participants to remember their code in the follow-up measure one year later. For the second measure point, each participating school was revisited by our research team that distributed and afterwards collected the second questionnaires. The sample was stratified by region, school type, and age with a total of *n* = 14 sampling units.

Participants and their caregivers were asked to provide written informed consent to participate voluntarily in the study. The project was approved by the local ethical commissions and corresponded to the Declaration of Helsinki.

The response rate of schools initially contacted amounted to 68%. Each assessment wave was conducted in the classrooms with supervision by trained psychologists. After data cleaning, incomplete data from 5.1% (*n* = 71) participants had to be eliminated leading to a final sample for wave 1 of *n* = 1313 adolescents. In wave 2, 75.3% (*n* = 989) of the original sample was successfully re-assessed. After data cleaning, a final sample with complete data for both waves of *n* = 941 was available for data analyses (cf. Table 1).

The only significant differences between adolescents lost to follow-up and those remaining in the sample concerned region and school type (each *p* < 0.001). In the category “small town” a higher percentage of adolescents were lost to follow up and the same was true for adolescents visiting lower secondary schools.

### 2.2. Measures

The Scale for the Assessment of Internet and Computer game Addiction (AICA-S; [29] is a validated self-report for the classification of Internet-related disorders. It is composed of the DSM-5-criteria for Internet Gaming Disorder (except for criterion deceiving). Each criterion (preoccupation, tolerance, withdrawal, continued use despite negative consequences, jeopardizing social or occupational relationships, mood regulation, and loss of control is assessed with one to two items on a 5-point Likert scale. In both, clinical and epidemiological research, AICA-S has demonstrated its diagnostic accuracy and psychometric properties [11,30].

For the assessment of personality traits, the Big-Five-Inventory-10 (BFI-10; [31] was administered. This short self-report is widely used and measures each domain (neuroticism, extraversion, openness, agreeableness, conscientiousness) of the Five-Factor Model with two items. For each trait factor, a mean score is calculated that is indicative for the specific manifestation of the trait. It has been demonstrated to be a valid and psychometrically sound questionnaire [31].

### 2.3. Classification of Symptom-Groups

In a first step we calculated a differential score for AICA-S at wave 1 and wave 2. Using visual classification with fixed cutoffs, we extracted 3 groups: Quitters (*n* = 154) were defined as adolescents showing a decrease in AICA-S from wave 1 to wave 2 (t1: M = 7.7, SD = 4.24; t2: M = 3.7, SD = 2.88). Accordingly, starters (*n* = 111) comprised adolescents showing a significant increase in AICA-S (t1: M = 3.2, SD = 2.25; t2: M = 9.5, SD = 3.62). Finally, a group of “stable-low” (*n* = 395) was extracted. This group consisted of adolescents showing a consistently low score of AICA-S below a threshold of 4.0 points (t1: M = 3.2, SD = 2.25; t2: M = 3.2, SD = 2.38). In order to validate these groups, we compared time spent online on a day of the weekend as an external criterion. For t1, a significant main effect for group was found (F = (2608) = 29.65, *p* = 0.001, η^2^ = 0.089) with a significant difference between quitters (M = 4.7, SD = 2.62) vs. starters (M = 2.9, SD = 2.40; *p* = 0.001) and quitters vs. stable-low (M = 3.0, SD = 2.40; *p* = 0.001) but not between starters and stable-low (*p* = 0.999). Likewise, for t2, a significant main effect was found (F = (2622) = 14.74, *p* = 0.001, η^2^ = 0.045) with significant differences between stable-low (M = 3.0, SD = 2.15) vs. starters (M = 4.3, SD = 2.20; *p* = 0.001) and quitters (M = 3.6, SD = 2.65) vs. starters (*p* = 0.020). No significant group-differences in demographics were found (cf. Table 2).

### 2.4. Statistical Analyses

For all analyses, SPSS 25 was used. Chi-square tests were used for categorical variables and *t*-tests for dependent and independent samples (with Cohen’s d as effect size measure) were calculated for the analyses of simple group differences.

To test predictive potential of personality traits at t1, multiple linear regression analyses were conducted to predict AICA-S score at t2. Sex and age, personality traits, and AICA-S-score at t1 were entered as separate models into the model. Additionally, moderated regression analyses were used to test changes of personality traits (t2–t1) as moderating factors. Here AICA-S-score at t1 was entered to predict AICA-S-score at t2. The Johnson–Neyman test of significant regions was used as follow-up-measure.

To examine developmental patterns of personality, separate two-factor ANOVAS were performed with personality traits as dependent variables for both measure points. IRD-course-groups (starters, quitters, stable-low) and sex were defined as factors. Partial eta-squared (η^2^) served as indicating the effect sizes and Games Howell test was used as a post hoc measure.

## 3. Results

### 3.1. Personality as a Predictor and a Moderator of Internet-Related Disorders

Stepwise multiple linear regression analyses with age, sex at step 1, personality traits at step 2, and baseline AICA-S score at step 3 were conducted on the score of AICA-S at t2. For step 2 (F(7) = 10.05, *p* = 0.001, R2 = 0.064, ∆R2 = 0.069) and step 3 (F(8) = 36.92, *p* = 0.001, R2 = 0.238, ∆R2 = 0.173) significant models were found. In step 2, conscientiousness (B = −0.89, SE B = 0.12, ß = −0.254, *p* = 0.001) and neuroticism (B = 0.23, SE B = 0.11, ß = 0.067, *p* = 0.047) were significantly related to AICA-S at t2. In step 3, AICA-S at t1 (B = 0.458, SE B = 0.03, ß = 0.461, *p* = 0.001) and conscientiousness (B = −0.291, SE B = 0.11, ß = −0.083, *p* = 0.009) yielded significance.

We were also interested in potential moderating effects of personality traits in the development of IRD. Thus, moderated regression analyses with baseline AICA-S score on AICA-S score at t2 and increments of personality traits (t2-t1) as moderators were performed. Openness was identified as a significant moderator B = −0.084; SE B = 0.03, *p* = 0.010; ∆R^2^ = 0.008. Johnson–Neyman test showed a significant effect (*p* < 0.05) for the range of openness between −3.03 and 3.31.

Furthermore, conscientiousness was identified as a significant moderator (B = −0.066; SE B = 0.03, *p* = 0.033; ∆R^2^ = 0.005). Johnson–Neyman test indicated a significant effect (*p* < 0.05) for the ranges between −3.36 and 3.72.

### 3.2. Mean-Level Changes of Personality and Their Dependence on Symptoms of Internet-Related Disorders

In order to determine differential developmental patterns in personality, two-factor-repeated measure ANOVAs were performed (cf. Table 3). As factors, IRD-course (quitters, starters, stable-low) and sex were entered; personality traits were the dependent variables. Main within-subject effects for personality were found for conscientiousness (*p* = 0.001; η^2^ = 0.040) and agreeableness (*p* = 0.001; η^2^ = 0.028). The within-subject interaction effects between personality and IRD-course were of particular interest. Here, interaction effects were found for conscientiousness (*p* = 0.001; η^2^ = 0.052) and neuroticism (*p* = 0.029; η^2^ = 0.011). For openness, a trend significant interaction was found (*p* = 0.070; η^2^ = 0.008). No interaction effects for sex were found.

In order to further analyze group-specific changes of these traits, *t*-tests for dependent samples were calculated. Among the quitters, neuroticism showed a significant decrease from t1 to t2 (*p* = 0.045, d = 0.18). Among the stable-low-group, significant decreases in conscientiousness occurred (*p* = 0.001, d = 0.15) and among starters, decreases in conscientiousness (*p* = 0.001, d = 0.66) and openness (*p* = 0.024, d = 0.23) were observable. Additionally, a trend significance for increasing neuroticism was found here (*p* = 0.063, d = 0.20).

**Figure 1:**
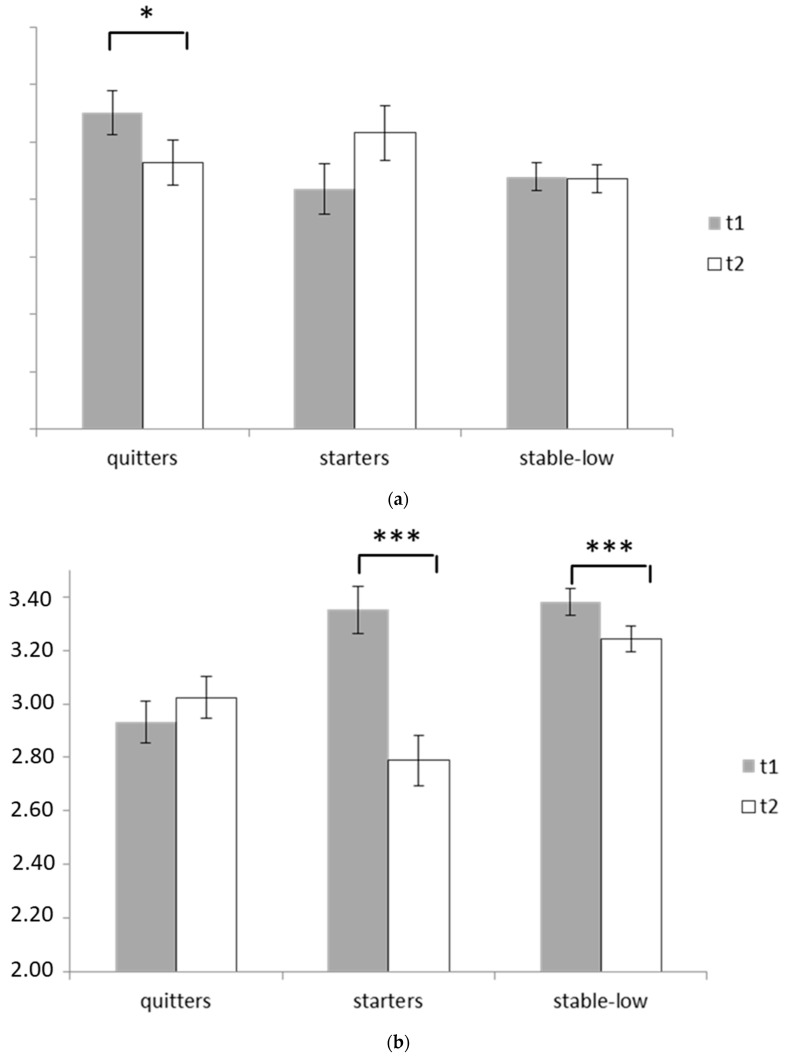
(**a**) Mean-level changes of neuroticism (*y*-axis) according to the course of symptoms of Internet-related disorders. (**b**) Mean-level changes of conscientiousness (*y*-axis) according to the course of symptoms of Internet-related disorders. * *p* > 0.05; *** *p* < 0.001.

The between-subjects comparisons displayed a significant effect of IRD course for conscientiousness only (F(2646) = 9.86, *p* = 0.001; η^2^ = 0.030). Here, significant differences between quitters vs. stable-low (t1: *p* = 0.001, d = 0.47; t2: *p* = 0.016, d = 0.24), between starters vs. stable-low (t2: *p* = 0.001, d = 0.51) and between starters vs. quitters (t1: *p* = 0.001, d = 0.43; t2: *p* = 0.037, d = 0.26) were found (cf. Figure 1a,b).

## 4. Discussion

The main purpose of our prospective study was to examine trait–pathology associations, specifically for the comparatively new phenomenon of Internet-related disorders in adolescents. As expected, personality traits were prone to slight changes in our sample. We found small decreases in agreeableness (η^2^ = 0.028) and conscientiousness (η^2^ = 0.040) which is in line with findings from prior research and reflects a general trend of personality development from early to mid-adolescence [26,31,32]. In addition, boys and girls differed regarding their personality traits with girls reporting higher scores than boys in each trait. Yet, sex had no influence on the development of personality over time, which matches prior findings [26].

We further hypothesized, that symptoms of IRD would affect the development of personality. Indeed, we found small to moderate effects on developmental patterns of personality depending upon the incidence or remission of IRD symptoms. These interaction effects encompassed particularly conscientiousness and neuroticism, and, to a lesser extent, openness. Adolescents whose Internet use became more excessive and was more closely related to symptoms of addictive use over the course of time, decreased in conscientiousness. In contrast, adolescents showing remission of IRD symptoms, showed small decreases in neuroticism. These results indicate that similar to findings from other addictive disorders [28], psychopathological symptoms influence the course of specific personality traits. Generally speaking, less psychopathological symptoms were related to enhancements in more functional traits (e.g., conscientiousness) while symptom incidence was accompanied by pronounced neuroticism, that can be regarded as a more dysfunctional trait related to a variety of mental health issues [33,34].

Conscientiousness has been related to facets like self-discipline and deliberation. It also incorporates elements of cognitive control, meaning that individuals high in this trait are able to resist immediate needs or temptations, and to delay gratification. Especially in adolescents, it has also been associated with better adjustment in school and a more accurate risk judgment that is preventive of risk-taking behavior [35,36]. Each aspect is relevant for IRD, since research has pointed out that impulsivity, decreased delay of gratification, and maladjustment in social settings are predisposing factors for seeking reward and comfort in virtual environments such as computer games or social media [37].

The importance of conscientiousness and neuroticism became again evident as both factors acted as predictors of IRD-symptoms one year later. This supports our hypothesis and findings from cross sectional studies [22,23,24]. While conscientiousness lowered the risk for IRD, the opposite was true for neuroticism. Conscientiousness also turned out as a moderating variable in the relationship between initial and later IRD. Changes in conscientiousness—and openness—were related to either an increased or decreased risk for later IRD. While increments in both factors were identified as protective factors, decrements had the opposite effect.

Openness has been described less frequently as a correlate of IRD. The factor represents (intellectual) curiosity and being sensible to feelings and values. Likewise, links towards a better regulation of stress have been reported [38]. One could speculate that adolescents high in openness or with increasing openness during maturation might feel the need to engage in things other than online games or virtual activities in the course of time. They could be less fascinated and satisfied by the formerly preferred online activity and thus turn their interest to alternatives which might represent a unique protective factor.

Our findings emphasize the complexity of the study on trait–pathology associations. On the one hand, we demonstrated that indeed some personality traits, namely conscientiousness and neuroticism can be perceived as factors increasing or decreasing the risk of later IRD symptoms. On the other hand, the same traits were sensitive of IRD symptoms and were prone to changes as a result of IRD. Accordingly, assumptions of contemporary etiological models for IRD were generally confirmed. Yet, we recommend adding assumptions to allow for a more precise depiction of not only causes but also consequences of IRD. Taking into account the moderating effects of conscientiousness but also of openness that has not yet been included in IRD-specific etiological models would add an interactive perspective to them. In addition, it would allow for having an idea for why some individuals seem to be able to spontaneously show remission of IRD symptoms. An explanation for this has been called for before [39,40]. It would also offer the opportunity of thinking of strategies of prevention and early intervention. Research has yielded some first evidence that personality traits can be changed by interventions [41]. Given that our results indicate that increases in conscientiousness and openness are indicators for decreasing IRD symptoms, one could think of specific strategies empowering both factors in adolescents, where externally driven trait maturation might be easier to initiate than in later life stages. 

With respect to our hypotheses, the findings lead to the following summary:

(1) We found a general, yet slight development of personality traits among adolescents over the course of one year
(2) Adolescents with IRD displayed different patterns in personality development compared to adolescents without IRD. Small to moderate effect sizes were found particularly for differential developmental paths of conscientiousness and neuroticism. Moreover, the changes in personality were partly associated with the stability of IRD-symptoms over the time.
(3) The moderated regression analyses further showed that openness and conscientiousness influenced the probability of displaying IRD-symptoms one year later. Thus, these traits can be perceived as important moderating variables.
(4) Finally, conscientiousness at baseline was revealed to be a direct predictor of IRD-symptoms one year later and thus might be understood as a risk factor.

Despite its novel features, our study has some limitations that need to be mentioned. First of all, our follow-up period amounted to only about one year. This might be too short for a fully reliable depiction of changes in personality traits that need time to develop. Moreover, as in many longitudinal surveys, a substantial percentage of adolescents were lost to follow-up reducing the statistical power of our analyses. Lastly, IRD is perceived as an umbrella concept, encompassing different addictive online behaviors like gaming or use of social media and pornography that might all be related to slightly differing predisposing factors and consequences. Unfortunately, it was not possible to distinguish between different IRD subtypes. Future research is needed here.

## 5. Conclusions

Taken together, our results show that some personality traits are both, directly related to IRD and likewise affected by symptoms of the addictive behavior. Interestingly, we found similar patterns of IRD-specific trait-pathology-associations for both genders which points out that etiological mechanisms are valid for boys and girls. Contrary to our expectations, we found no associations with extraversion that has been discussed as a correlate for IRD before. Yet, as a novel finding, we found that maturation of openness should be taken into consideration as a factor of potential interest in IRD.

Particularly with regards to prevention and early intervention of IRD among minors the findings have some practical implications. As stated above, there is evidence that the normative development of personality traits can be stimulated by specific techniques. For instance, behavioral activation [41] can be considered as one strategy appropriate for fostering functional facets of traits (e.g., social assertiveness, self-organization skills) and likewise regulating dysfunctional aspects of others (e.g., anxiety, responsiveness towards stress). According to behavioral activation and theoretical concepts like the expectancy value theory, functional traits can be emphasized by identifying activities, interests, and everyday routines in line with personal values. There is first evidence that a guided engagement in such behaviors can support a functional personality maturation and therefore might be a useful prevention strategy against IRD. However, these assumptions need to be evaluated in future research activities.

## Figures and Tables

**Table 1:** Sociodemographic and baseline variables for adolescents attending the first measure point and adolescents being successfully recruited at the second measure point.

Sociodemographics and Baseline Variables	Wave 1 (*n* = 1313)	Wave 2 (*n* = 941)
**Sex; *n* (%)**		
female	701(53.4)	520 (55.3)
male	612 (46.3)	421 (44.7)
**School type; *n* (%)**		
Lower secondary and secondary	102 (7.8)	82 (8.7)
Integrated, comprehensive	270 (20.6)	215 (22.8)
High school	941 (71.7)	644 (68.4)
**Migration background; *n* (%)**		
Yes	43 (3.3)	33 (3.5)
no	1214 (92.5)	878 (93.3)
Missing	56 (4.3)	30 (3.2)
**Residence (region); *n* (%)**		
rural	497 (37.8)	392 (41.7)
small town	444 (33.8)	237 (25.2)
city	372 (28.3)	312 (33.2)
**Baseline measures; *M* (*SD*)**		
AICA-S	3.9 (3.29)	4.5 (3.23)
BFI: Neuroticism	2.9 (0.96)	2.9 (0.99)
BFI: Extraversion	3.5 (0.88)	3.5 (0.97)
BFI: Openness	3.5 (1.02)	3.4 (1.03)
BFI: Agreeableness	3.3 (0.84)	3.2 (0.84)
BFI: Conscientiousness	3.3 (0.92)	3.1 (0.91)

Note: *M* = mean; *SD* = standard deviation; AICA-S = Scale for the Assessment of Internet and Computer game Addiction; BFI = Brief Five Factor Inventory.

**Table 2:** Sociodemographic description of adolescents with occurring and remitting symptoms of Internet-related disorders and stable non-problematic users.

Sociodemographics	Course of IRD-Symptoms According to AICA-S			Statistical Comparison
	Stable-Low (*n* = 395)	Quitters (*n* = 154)	Starters (*n* = 111)	
**Age; M (SD)**	13.1 (1.03)	13.1 (1.05)	12.9 (1.06)	*p* = 0.343
**sex; *n* (%)**				
Female	226 (57.2)	94 (61.0)	66 (59.5)	*p* = 0.698
**school type; *n* (%)**				*p* = 0.195
Lower secondary and secondary	37 (9.4)	19 (12.3)	8 (7.2)	
Integrated, comprehensive	82 (20.8)	36 (23.4)	33 (29.7)	
High school	276 (69.9)	99 (64.3)	70 (63.1)	
**migration background; *n* (%)**				*p* = 0.147
yes	8 (2.1)	6 (3.9)	6 (5.6)	

Note: *n* = 666; *M* = mean; *SD* = standard deviation; *p* = level of significance; IRD = Internet-related disorders; AICA-S = Scale for the Assessment of Internet and Computer game Addiction; starters = adolescents with increments in symptoms of Internet-related disorders at t2; quitters = adolescents with decrements in symptoms of Internet-related disorders at t2; stable-low = adolescents without symptoms of Internet-related disorders at t1 and t2.

**Table 3:** Mean-level changes of personality traits over time under consideration of symptoms of Internet-related disorders.

Personality Traits and Cluster	Assessments (Time)		(1) Main effect
			(2) Interaction effect
	t1 *M* (*SD*)	t2 *M* (*SD*)	
**Neuroticism**			
Starters	2.84 (0.93)	3.03 (1.01)	(1) *n.s.* (2) *F*(2642) = 3.56, *p* = 0.029; η^2^ = 0.011
Quitters	3.09 (0.96) ^a^	2.93 (0.97) ^b^	
Stable-low	2.88 (0.99)	2.87 (0.97)	
**Extraversion**			
Starters	3.53 (0.86	3.59 (0.94	(1) *n.s.* (2) *n.s.*
Quitters	3.46 (0.95)	3.43 (1.01)	
Stable-low	3.41 (0.90)	3.43 (0.98)	
**Openness**			
Starters	3.52 (0.90) ^a^	3.29 (1.08) ^b^	(1) *n.s.* (2) *F*(2645) = 2.67, *p* = 0.070; η^2^ = 0.008
Quitters	3.40 (1.06)	3.48 (1.02)	
Stable-low	3.54 (0.99)	3.52 (1.00)	
**Agreeableness**			
Starters	3.37 (0.92)	3.08 (0.83)	(1) *F*(1647) = 18.63, *p* = 0.001; η^2^ = 0.028 (2) *n.s.*
Quitters	3.29 (0.80)	3.18 (0.87)	
Stable-low	3.40 (0.82)	3.24 (0.82)	
**Conscientiousness**			
Starters	3.35 (0.90) ^a^	2.79 (0.80) ^b^	(1) *F*(1646) = 26.84, *p* = 0.001; η^2^ = 0.040 (2) *F*(2646) = 17.59, *p* = 0.001; η^2^ = 0.052
Quitters	2.93 (1.00)	3.02 (0.96)	
Stable-low	3.38 (0.91) ^a^	3.24 (0.90) ^b^	

Note. N = 666; t1 = wave 1; t1 = wave 2; *M* = mean; *SD* = standard deviation; *F* = *F*-value (ANOVA) with degrees of freedom in brackets; *p* = *p*-value (level of significance); η^2^ = partial eta-square (effect size); ^a,b^: different superscripts indicate significant within-subject differences (*p* ≤ 0.05); starters = adolescents with increments in symptoms of Internet-related disorders at t2; quitters = adolescents with decrements in symptoms of Internet-related disorders at t2; stable-low = adolescents without symptoms of Internet-related disorders at t1 and t2.

## Data Availability

Not applicable.
